# Genotype-Phenotype Correlation and Psychiatric Manifestations in a Case of Phelan-McDermid Syndrome With 22q13.33 Deletion

**DOI:** 10.7759/cureus.92379

**Published:** 2025-09-15

**Authors:** Mario Alberto Puente Torres, Juan Jorge Palacios Casados, Yvonne Flores Medina, Aideé Gonzalez Gutierrez, Carlos Sabás Cruz Fuentes

**Affiliations:** 1 Department of Psychiatric Genetics, Ramón de la Fuente Muñiz National Institute of Psychiatry, Mexico City, MEX; 2 Department of Clinical Research, Ramón de la Fuente Muñiz National Institute of Psychiatry, Mexico City, MEX; 3 Department of Clinical Neuropsychology, Faculty of Higher Studies Iztacala, National Autonomous University of Mexico, Tlalnepantla, MEX

**Keywords:** 22q13.33 deletion, bipolar disorder, cognitive-behavioral aspects, genomic medicine, intellectual disability, neuropsychiatric symptoms, personalized medicine, phelan-mcdermid syndrome, ring chromosome 22, shank3 gene

## Abstract

Phelan-McDermid syndrome (PMS) (OMIM: 606232) is a rare genetic disorder usually caused by deletions in the 22q13.3 region, often affecting the *SHANK3* gene, which is essential for synaptic function and neurodevelopment. PMS is clinically heterogeneous, presenting with intellectual disability, language deficits, hypotonia, and psychiatric conditions such as autism spectrum disorder and bipolar disorder.

We present the case of a 29-year-old woman with intellectual disability, language impairment, and bipolar disorder. She was initially diagnosed with a ring chromosome 22, and subsequent microarray analysis revealed a pathogenic deletion involving *SHANK3*. Clinically, she displayed affective instability, recurrent psychotic episodes, multiple psychiatric hospitalizations, facial dysmorphisms, scoliosis, and an increased pain threshold.

This case illustrates the importance of integrating genetic and psychiatric evaluations in patients with intellectual disability who present with early-onset psychiatric symptoms. Timely recognition of psychiatric manifestations in PMS has direct clinical implications, as early interventions may improve outcomes. Furthermore, the coexistence of a ring chromosome 22 and a 22q13.3 microdeletion highlights the complexity of genotype-phenotype correlations in PMS and suggests that genes beyond *SHANK3* may influence neurodevelopmental and psychiatric features.

## Introduction

The microdeletion syndrome 22q13.3, or Phelan-McDermid syndrome (PMS) (OMIM: 606232), has an estimated occurrence rate of 2.5-10 cases per one million births [1]. Diverse genetic alterations are associated with the syndrome, most commonly affecting individuals carrying a deletion in the long arm of chromosome 22. Other alterations include an unbalanced translocation or a ring chromosome. In the latter, the prevalence is 1/1,000,000. In any case, the size of the deleted segments ranges from over 100 kb to more than nine Mb. Interestingly, there is a relationship between the deletion size and the severity of the syndrome [2].

The identified critical genomic region contains the *ACR, RABL2B*, and *SHANK3* genes. The expression of the latter produces a postsynaptic density scaffolding protein (SHANK3) located in excitatory synapses with multiple roles, interfacing with ion channel receptors, signaling molecules, and cytoskeletal proteins. Moreover, it is involved in the regulation of dendritic spine maturation [3]. A pathogenic variant in the *SHANK3* gene has been reported to be linked to PMS [4]. Overall, *SHANK3* has a fundamental role in neuropsychiatric and neurodevelopmental functions [5]. Clinically, its haploinsufficiency is characterized by neonatal hypotonia, recurrent upper airway infections, increased sensitivity to sensory stimuli, congenital anomalies, diverse developmental abnormalities, minor dysmorphic facial features, impaired speech, and affected expressive language [6]. While much attention has focused on the neurodevelopmental and somatic aspects of PMS, psychiatric manifestations are frequent yet often underrecognized, highlighting the need to broaden the clinical phenotype to include affective and cognitive disturbances.

A cohort study reported that approximately half of the patients who bore small deletions in the 22q13.3 region manifested affective and cognitive psychiatric symptoms [7]. These mood and behavioral oscillations are prominent in young adults and could increase the risk of developing bipolar disorder. Moreover, the dysfunctional modulation of cognition and emotion contributes to the heightened sensitivity to environmental stimuli that could exacerbate emotional fluctuations and behavioral changes [8]. Early recognition of psychiatric features in PMS is clinically significant, as it allows timely interventions that may improve long-term outcomes.

## Case presentation

A 29-year-old woman was referred to the Department of Genetics for evaluation due to a diagnosis of intellectual disability and bipolar disorder. The proband came from a nonconsanguineous family with phenotypically normal parents, without a history of hereditary diseases. No significant pathological findings during prenatal care that could be related to intellectual disability were reported.

Due to the breech presentation, the proband was born at term via cesarean section; however, no signs suggestive of fetal distress, abnormalities, or signs of hypotonia were reported at birth. At two years and six months old, her parents identified a language developmental delay, although other neurodevelopmental milestones appeared unaffected. She underwent language therapy, and the diagnoses of anaphasic and anarthrous language development delay were made. Therapy was maintained until the age of six.

In addition, she underwent a neuropsychological evaluation at nine, obtaining a global IQ of 61. Notably, her lowest score was in the language assessment subscale. It was also at this time that hypotonia and gait abnormalities were identified. Her medical history also reported multiple upper respiratory tract infections during childhood. At this stage, the patient also had her first assessment by a child psychiatrist, receiving a diagnosis of attention-deficit/hyperactivity disorder. Treatment with methylphenidate was initiated and continued for two years.

**Figure 1:**
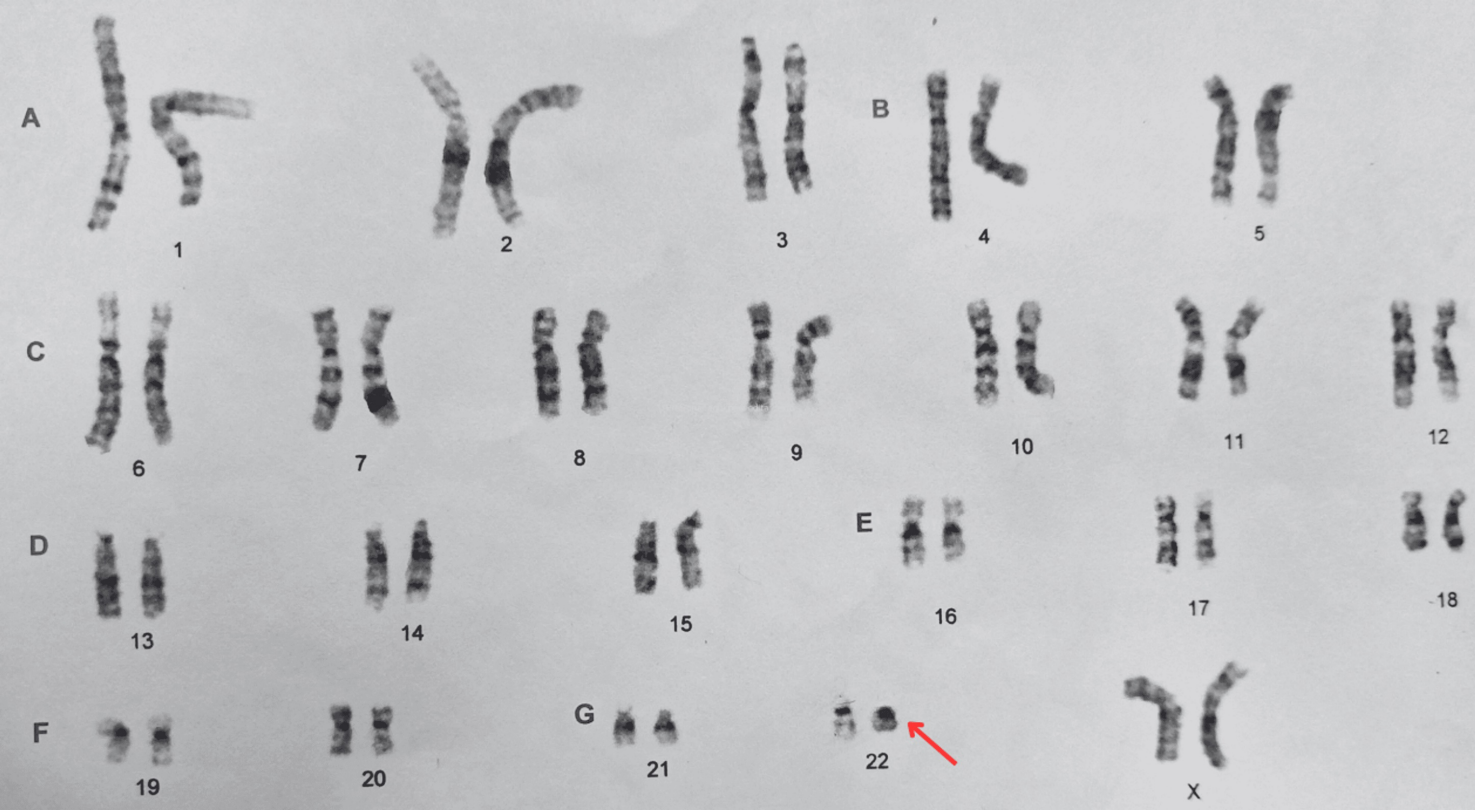
Chromosomes karyotype result 46,XX,r(22)(p11q13) The arrow points to the presence of the ring chromosome 22

Due to developmental abnormalities, at the age of 12, a referral to a medical genetics service was suggested. The service requested a conventional karyotype analysis in peripheral blood using G-banding at a resolution of 500 bands, identifying a 46, XX,r(22)(p11q13) karyotype, suggesting a microdeletion syndrome (Figure 1). Therefore, additional studies were suggested to define the breakpoint regions.

At the age of 15, due to emotional dysregulation triggered by the loss of a family member, she was reassessed by a psychiatrist. This episode was characterized by verbal and physical hetero-directed aggression toward family members. Moreover, she ran away from home during this episode and was missing for three days before returning. Upon her return, she was taken to the psychiatric emergency service, requiring hospitalization for 20 days, when new psychiatric symptoms emerged, including unusual thoughts and erratic behavior. These findings led to a diagnostic reevaluation, with a provisional diagnosis of a psychotic disorder under study to rule out schizophrenia. Treatment with risperidone and biperiden was initiated; however, because of the development of an incapacitating rigidity, a switch to magnesium valproate was required. The absence of a clinical improvement led to a subsequent change to fluoxetine, with a good response. 

At 16, a new stressor, the loss of another family member, triggered a new crisis characterized by predominant depressive symptoms requiring psychiatric care in a private hospital, where fluoxetine was discontinued and sertraline was initiated. Four months later, she experienced another episode of psychiatric deterioration, this time with predominant manic and psychotic symptoms, requiring hospitalization for 25 days. Since a chronic psychotic disorder diagnosis was suggested, pharmacotherapy with risperidone, biperiden, and magnesium valproate was resumed, apparently leading to remission of both mania and psychosis. Given the stability of her symptoms, her treatment was transitioned again to fluoxetine, which was maintained for eight years with good affective stability. She was eventually discharged from private psychiatric care without further pharmacological treatment.

In 2022, at the age of 28, she experienced episodes characterized by sleep pattern disturbances (decreased need for sleep), increased energy levels, and psychomotor agitation. She also exhibited physical hetero-directed aggression toward her mother, leading to her admission to the psychiatric emergency service at the Instituto Nacional de Psiquiatría. Treatment with risperidone was initiated, requiring dose adjustments due to daytime drowsiness. A month later, she experienced a recurrence of symptoms, leading to an increase in her dosage. Since bipolar disorder was suspected, she was referred to the Affective Disorder Clinic, where the diagnosis was confirmed based on the Diagnostic and Statistical Manual of Mental Disorders (DSM-5) criteria. Therefore, her pharmacological management includes valproic acid and risperidone, which continues to date.

**Figure 2:**
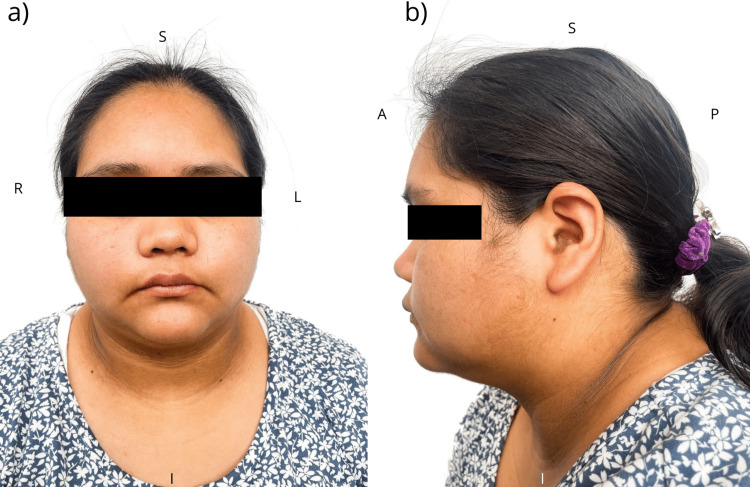
Phenotypic characteristics in a patient Photographs showing the frontal (a) and lateral (b) views of the patient, highlighting key phenotypic features relevant to the clinical presentation and diagnosis. The patient presents with a bulbous nasal tip, mild nasal septum deviation, facial asymmetry, prominent supraorbital ridges, and acanthosis. Orientation: frontal view-superior (S), inferior (I), patient’s right (R), patient’s left (L). Lateral view-superior (S), inferior (I), anterior (A), posterior (P)

**Figure 3:**
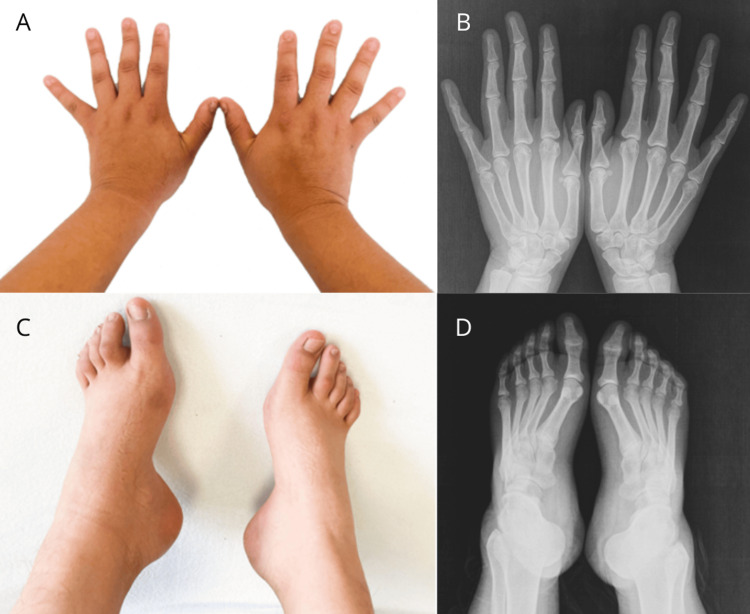
Bilateral cavus foot and camptodactyly with lateral deviation of the distal phalanges X-ray images confirming bilateral cavus foot and camptodactyly with lateral deviation of the distal phalanges of the middle fingers in both hands. (A-B) Hands in dorsopalmar view, showing camptodactyly with lateral deviation of the distal phalanges of both middle fingers. (C-D) Bilateral cavus feet, shown in dorsal view; radiographs obtained in anteroposterior (AP) view

**Figure 4:**
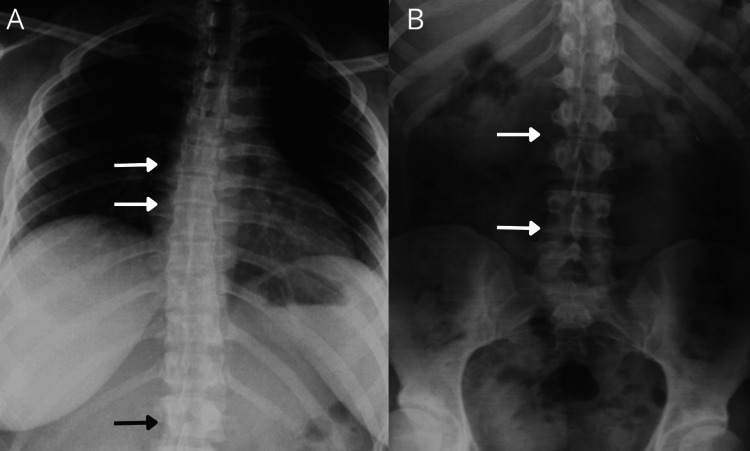
X-ray images of the spine with highlighted areas of curvature X-ray images showing mild scoliosis and pelvic asymmetry. (A) Dorsal spine in anteroposterior (AP) view, showing mild right convex scoliosis. (B) Lumbar spine in AP view, showing mild left convex scoliosis. White arrows indicate the regions of abnormal curvature

At this stage, upon reviewing previous genetic studies, the clinician requested a new evaluation by the clinical genetics service. Physical examination rendered the following phenotype: bulbous nasal tip, mild nasal septum deviation, facial asymmetry, prominent supraorbital ridges, dental malocclusion, acanthosis (Figure 2), bilateral cavus foot, and camptodactyly with lateral deviation of the distal phalanges of the middle fingers in both hands, confirmed by X-ray (Figure 3). Additionally, sacral hyperlordosis was observed, confirmed by spinal X-ray (Figure 4), and the absence of pain perception. These clinical features (Table 1), combined with the patient´s clinical history and previous karyotype results, suggested a diagnosis of PMS, prompting a more detailed diagnostic approach.

**Table 1:** Phelan-McDermid syndrome features present in our patient Clinical features of Phelan-McDermid syndrome (22q13.3 deletion) in the evaluated patient. Features present are listed in the left column, and features absent are listed in the right column

Present in the patient	Absent in the patient
Hypotonia	Sleep disturbance
Increased tolerance to pain	Febrile/nonfebrile seizure
Clinodactyly of the fifth finger	Brain abnormalities
Kyphoscoliosis	Large, fleshy hands
Bulbous nasal tip	Syndactyly of 2nd and 3rd toes
Alterations in the ears	Hyperextensibility
Periorbital fullness	Dimple in sacrum
Malocclusion/separated teeth	Short stature/growth retardation
Malar hypoplasia	Tall stature/accelerated growth
Midface hypoplasia	Microcephaly
Low-set ears	Dolichocephaly
Acanthosis	Macrocephaly
Constipation/diarrhea	Epicanthal folds
Recurrent respiratory infection	Hypertelorism
	Sunken eyes
	Wide nasal bridge
	Long philtrum
	High narrow palate
	Micrognathia
	Prominent lips
	Prominent cheeks
	Eyelid ptosis
	Sparse / spiral hair
	Gastroesophageal reflux
	Congenital heart disease
	Kidney disorders
	Lymphedema
	Precocious or delayed puberty
	Exotropia/strabismus
	Dysplastic/hypoplastic nails
	Hypothyroidism

Complementary studies, including brain magnetic resonance imaging, ophthalmologic examination, echocardiogram, hemogram, blood chemistry, and thyroid function tests, did not show further abnormalities.

A neuropsychological evaluation conducted to evaluate general intellectual capacity, language, perception, memory, executive function, functional capacity, personality, and behavior ruled out autism spectrum disorder (ASD) or ADHD diagnoses. However, the patient obtained an intelligence quotient of 55, with significant impairment in language, particularly in phoneme discrimination and auditory-to-verbal information transcoding. The impairment in tactile stimulus recognition leads to a diagnosis of amorphognosia.

**Figure 5:**
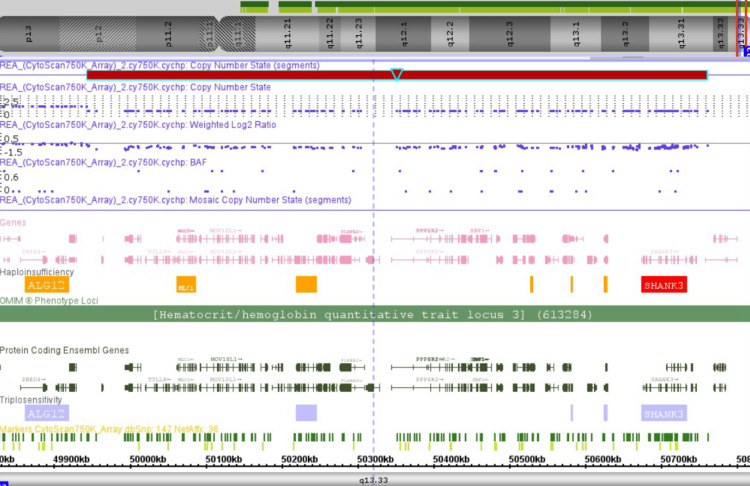
Chromosomal microarray showing microdeletion on chromosome 22 Chromosomal microarray analysis revealing a heterozygous deletion in the long arm of chromosome 22: arr[hg38] 22q13.33(49942377_50759338)x1. This deletion spans 817 kbp and includes 28 OMIM-listed genes, with *SHANK3* being among the most relevant

A DNA microarray analysis using CytoScan 750k (Affymetrix Thermo Fisher) revealed an 817 kbp deletion in the 22q13.33 region of the long arm of chromosome 22 (arr[hg38]22q13.33(49942377_50759338)x1) (Figure 5). The deletion encompasses 28 genes registered in OMIM, including *SHANK3*. These findings substantiated the diagnosis of PMS.

## Discussion

We describe here the clinical and molecular findings of a patient with bipolar disorder, intellectual disability, and facial dysmorphisms. A deletion of 817 kbp encompassing 28 genes was detected (Supplementary Table 2). Although parental karyotypes were analyzed due to the ring chromosome 22 in the proband, no chromosomal anomalies were seen in either parent, a result consistent with de novo deletions reported in the literature [1,5].

Genotype-phenotype correlation studies have associated the size of the deletion with specific clinical features in patients; however, this relationship is not uniform. Among the most frequently reported manifestations are neurological disorders such as delayed speech development, neurodevelopmental disorders, and psychiatric disorders, including ASD, ADHD, schizophrenia, and bipolar disorder.

These phenotypic variations may be partially explained by the contribution of specific genes within the deleted region [9]. Nevertheless, significant variability has been documented, even among individuals with deletions of identical size, and the phenotype is therefore inconsistent across reported cases [1,8].

The phenotype of our patient overlaps with findings described in other cohorts, with the most significant clinical features including intellectual disability, delayed language development, hand and foot malformations, analgesia, facial dysmorphia, and scoliosis [4]. The psychiatric symptoms debuting in early adolescence in our patient are consistent with a report in which 71% of PMS-affected individuals presented symptom onset between the ages of nine and 20 [10].

Besides the archetypal *SHANK3* gene, the loss of additional genes included in the 817 kbp deletion, such as *MLC1, PLXNB2,* *SCO2*, and *MAPK8IP2*, may also contribute to the patient’s phenotype. Importantly, previous studies suggest that larger deletions encompassing multiple genes beyond *SHANK3* are often associated with more complex clinical presentations, indicating that both the extent of the deleted region and the cumulative effect of the disrupted genes may help explain the severity of the observed phenotype [2].

Among these genes, *MLC1* has been implicated in susceptibility to schizophrenia and bipolar disorder due to its role in neuromodulation; decreased expression has also been linked to various psychiatric manifestations [11]. *PLXNB2* may provide neuronal protection, particularly in stress response pathways dysregulated in mood disorders, and has been shown to regulate peripheral somatosensory neurons and nociception, which may be relevant to proprioceptive anomalies and the increased pain threshold observed in this patient [12]. *PLXNB2* is also involved in osteogenic differentiation, and its dysregulation could be related to the bone manifestations of the syndrome [13]. Another gene of interest is *SCO2*, essential for nervous system development, where dysregulation may contribute to intellectual disability [14]. In animal studies, haploinsufficiency of *MAPK8IP2* has been associated with locomotor deficits, ataxic gait, impaired social interactions, learning difficulties, and defective neuronal maturation [12].

*SHANK3* itself is involved in multiple developmental functions and is considered critical for this syndrome, with potential contributions to behavioral and neurodevelopmental disorders as well as increased risk of schizophrenia, bipolar disorder, catatonia, and neuropsychiatric regression [15]. Beyond *SHANK3*, the deletion encompasses genes converging on interconnected biological pathways: *MAPK11, MAPK12*, and *MAPK8IP2* in MAP kinase-mediated neuronal signaling; *MLC1* and *PANX2* in ion homeostasis and intercellular communication; *SCO2*, *SELENOO*, and *CPT1B* in mitochondrial bioenergetics; and HDAC10 and NCAPH2 in chromatin remodeling and cell cycle regulation. Taken together, this gene network may underlie synaptic dysfunction, impaired mitochondrial function, and altered cytoskeletal organization, thereby providing a mechanistic framework for the neurological severity observed in this case [3].

The ring chromosome identified in the proband has been associated with an increased predisposition to bipolar disorder. In one report, more than half of PMS patients met criteria for this condition [16]. Interestingly, bipolar disorder has been more frequently reported in patients with deletions smaller than 1 Mb [17]. However, psychopathology overall occurs in only 5% of PMS patients and appears to arise almost exclusively when the chromosomal break is near the terminal end [18].

Treatment in such cases is complex due to the limited efficacy and poor tolerability of standard therapies [8]. Our patient exhibited resistance to multiple medications, consistent with previous reports indicating reduced antidepressant effectiveness and partial improvement with combined use of antipsychotics and anticonvulsants, ultimately leading to gradual stabilization of mood and behavior [4,11].

## Conclusions

PMS is characterized by significant phenotypic variability. Due to its overlap with other conditions, its clinical diagnosis is a challenge, contributing to its underdiagnosis. This case highlights the importance of a multidisciplinary approach that requires systematic evaluations of clinical features, genetic testing, and neuropsychology assessments to achieve an accurate diagnosis. The diagnostic confirmation through molecular analysis, identifying a deletion in 22q13.3,3, establishes a probable direct correlation between this genetic alteration and the patient´s clinical presentation. However, this case also underscores the need to investigate the role of other genes besides the *SHANK3* within the deleted region, as they may better explain the phenotypic variability and aid in optimizing clinical management. Additionally, it emphasizes the importance of incorporating molecular testing in patients with intellectual disability and other psychiatric disorders, as has been done for autism spectrum disorders.

A recurring negative aspect in the routine evaluation of patients with genetic syndromes has been the lack of consistency of psychiatric and neuropsychological testing to identify early risk symptoms that, as in this case, eventually led to being expressed as a bipolar disorder, thus delaying appropriate clinical management. Moreover, this case underscores the relevance of personalized pharmacology treatment tailored to the patient's specific needs to improve his quality of life and promote better clinical outcomes. It reinforces the necessity of establishing a multidisciplinary team in the management of patients with intellectual disabilities and genetic syndromes. Future research should focus on refining genotype-phenotype correlation to develop targeted therapeutic strategies based on neuropsychological and medical needs, identifying risk factors associated with poor prognosis, and providing appropriate genetic counseling. These approaches would improve the diagnostic process and the patient´s quality of life with PMS.

## Appendices

**Table 2:** OMIM genes deleted in the 817 kbp 22q13 region in this patient List of 28 OMIM genes deleted in the 817 kbp region of chromosome 22q13 in the patient. The deletion includes SHANK3 and other genes potentially contributing to the observed neurodevelopmental and psychiatric phenotype

Gene symbol	Full name	Summary/function
PIM3	Pim-3 Proto-Oncogene, Serine/Threonine Kinase	Ser/Thr kinase family; overexpressed in hematological and epithelial tumors; associated with MYC coexpression; regulates signal transduction, cell proliferation, and survival
IL17REL	Interleukin 17 Receptor E Like	Predicted interleukin-17 receptor activity; involved in cytokine-mediated signaling; extracellular localization
TTLL8	Tubulin Tyrosine Ligase Like 8	Protein-glycine ligase activity; roles in axoneme assembly, sperm motility, and cilium assembly
MLC1	Modulator Of VRAC Current 1	Putative membrane transporter; mutations cause megalencephalic leukoencephalopathy with subcortical cysts (autosomal recessive)
MOV10L1	Mov10 Like RNA Helicase 1	Testis-specific RNA helicase; multiple isoforms
PANX2	Pannexin 2	Gap junction component in CNS; co-expressed with pannexin 1 in neurons; forms distinct cell-type specific junctions
SELENOO	Selenoprotein O	Largest mammalian selenoprotein, localized to mitochondria; contains selenocysteine; likely involved in redox regulation
TUBGCP6	Tubulin Gamma Complex Component 6	Component of centrosomal γ-tubulin complex; essential for microtubule nucleation; associated with microcephaly and chorioretinopathy
HDAC10	Histone Deacetylase 10	Histone deacetylation; regulates chromatin, transcription, and cell cycle; associated with neuroblastoma and gaze palsy
MAPK12	Mitogen-Activated Protein Kinase 12	Stress-activated MAP kinase; involved in myoblast differentiation; linked to breast cancer
MAPK11	Mitogen-Activated Protein Kinase 11	MAP kinase activated by cytokines/stress; regulates cell proliferation and differentiation; linked to disorders of sexual development
PLXNB2	Plexin B2	Semaphorin receptor; roles in axon guidance and cell migration; linked to amelogenesis imperfecta and spinocerebellar ataxia
DENND6B	DENN Domain Containing 6B	Guanyl-nucleotide exchange factor; localized to cytosol/recycling endosome
PPP6R2	Protein Phosphatase 6 Regulatory Subunit 2	Regulatory subunit of protein phosphatase-6; essential for mitotic spindle formation and chromosome alignment
SBF1	SET Binding Factor 1	Pseudophosphatase with GEF domain; mutations cause Charcot-Marie-Tooth disease type 4B3
ADM2	Adrenomedullin 2	Hormone regulating cardiovascular homeostasis, prolactin release, fluid balance; linked to coronary stenosis
MIOX	Myo-Inositol Oxygenase	Catalyzes inositol catabolism; involved in metabolism; linked to pigmentation disorders
NCAPH2	Non-SMC Condensin II Complex Subunit H2	Regulatory condensin-2 complex subunit; essential for mitotic chromosome condensation and neurogenesis
SCO2	Synthesis Of Cytochrome C Oxidase 2	Metallochaperone required for cytochrome c oxidase biogenesis; mutations cause infantile encephalocardiomyopathy
TYMP	Thymidine Phosphorylase	Angiogenic factor; mutations cause mitochondrial neurogastrointestinal encephalomyopathy
KLHDC7B	Kelch Domain Containing 7B	Protein with Kelch domain; linked to autosomal recessive deafness
SYCE3	Synaptonemal Complex Central Element Protein 3	Involved in meiotic recombination and spermatogenesis; mutations cause spermatogenic failure
CPT1B	Carnitine Palmitoyltransferase 1B	Controls fatty acid β-oxidation in muscle mitochondria; mutations cause CPT1 deficiency
CHKB	Choline Kinase Beta	Catalyzes first step in phosphatidylcholine biosynthesis; mutations cause congenital muscular dystrophy (megaconial type)
MAPK8IP2	Mitogen-Activated Protein Kinase 8 Interacting Protein 2	Scaffold protein for JNK signaling; linked to spinocerebellar ataxia and carcinomas
ARSA	Arylsulfatase A	Lysosomal enzyme; mutations cause metachromatic leukodystrophy (progressive demyelination disorder)
SHANK3	SH3 And Multiple Ankyrin Repeat Domains 3	Postsynaptic scaffold protein; mutations linked to autism spectrum disorder and Phelan-McDermid syndrome
ACR	Acrosin	Serine protease in sperm acrosome; essential for penetration of zona pellucida during fertilization
